# P91 - Pilot study on sensitisation profiles of children with a primary tree nut allergy

**DOI:** 10.1186/2045-7022-4-S1-P146

**Published:** 2014-02-28

**Authors:** Nanna Juel-Berg, Kirsten Skamstrup Hansen, Lars Kaergaard Poulsen

**Affiliations:** 1 Allergy Clinic, Copenhagen University Hospital-Gentofte, Gentofte, Denmark; 2 Children’s Medical Center, CUH-Herlev, Herlev, Denmark

## Background

Plant food allergy is a relatively frequent medical problem in childhood. Peanut and tree nuts are the most common causes. Some are allergic to tree nuts due to pollen cross reactivity whereas others have a primary tree nut allergy.

Cross sensitivity is also seen between tree nuts and is serologically well described. But the clinical picture of cross sensitivity amongst tree nut allergic children is less investigated.

## Aim

We aim to investigate sensitization patterns and clinical reactions of the most common and clinical relevant tree nut allergies in Denmark. We want to explore this profile in children and young adults suspected of having a primary tree nut allergy.

This pilot study will be used as a guide for a study with a larger set up including controlled oral food challenges (OFCs) to confirm allergy or ensure tolerance.

Our aim is to recruit 40-50 and each participant shall undergo OFCs with four nuts.

## Methods

Children and young adults (age 0-23) with sensitization to tree nuts were sought out in our IgE (ImmunoCAP®) database.

Medical files of patients with IgE-levels to hazelnut > IgE to birch pollen OR with positive (>0.35 kIU/L) specific IgE to any other tree nut were identified. These selection criteria were used as indications for a primary tree nut allergy.

Medical files on 28 children ages 0-16 were randomly selected. Medical history including prior exposure and allergic reactions to plant foods and tree nuts was obtained from the files. Inclusion criteria were the primary indications together with a medical history of an allergic reaction to a tree nut.

## Results

• 197 patients had IgE-levels to hazelnut > IgE to birch pollen OR another positive specific IgE to a tree nut.

• Of the 28 screened 19 had a medical history with at least one allergic reaction to tree nuts.

• Out of the above mentioned 19 children n=x had a history with a positive reaction (ranging from mild to severe symptoms) to the following plant foods; Hazelnut (n=14), Peanut (n=8), Cashew (n=3), Pistachio (n=5), Almond (n= 2), Walnut (n=7), Coconut (n=1), Sesame (n=2), Poppy seed (n=2), Sunflower seed (n=1), Soy (n=1), Pine nut (n=1).

## Conclusion

This study suggests that we can expect that n =133 patients attending our clinics are eligible for the OFC project.

Deducted form the medical records hazelnut, walnut, pistachio and cashew were the four most common tree nuts involved in prior allergic reactions.

